# Burnout Among Surgeons in the UK During the COVID-19 Pandemic: A Cohort Study

**DOI:** 10.1007/s00268-021-06351-6

**Published:** 2021-10-26

**Authors:** Jonathan Houdmont, Prita Daliya, Elena Theophilidou, Alfred Adiamah, Juliet Hassard, Dileep N. Lobo, the East Midlands Surgical Academic Network (EMSAN) Burnout Study Group, Jamil Ahmed, Victor Babu, Daryll Baker, David Bartlett, Ian Beckingham, Imran Bhatti, Adam Brooks, Steven Brown, Josh Burke, Hannah Byrne, Ian Chetter, Hannah Cook, James Coulston, Lucinda Cruddas, Richard Dias, Frank Dor, Mukul Dube, Katherine Grant, John Hammond, Rachel Hargest, Theophilus Joachim, Annie Joseph, Naveed Kara, Dimitrios Karavias, Sita Kotecha, Anisa Kushairi, Roshan Lal, Kit Lam, Irwin Lasrado, Rachel Lee, Gurminder Mann, George Mannu, Charles Maxwell-Armstrong, James McCaslin, Frank McDermot, Andrew Miller, Sarah Miller, Jenna Morgan, Sandip Nandhra, Sangara Narayanasamy, Patrick O’Brien, Laura Parry, Kizzie Peters, Marina Pitsika, Emmanouil Psaltis, Kevin Sargen, Panchali Sarmah, Murali Shyamsundar, Chinnappa Reddy, Katie Rollins, Farah Roslan, Joseph Shalhoub, Matt Stanislas, Benjamin Tan, Nilanjana Tewari, Pradeep Thomas, Tony Thomas, Jim Tiernan, Giles Toogood, Karl Trimble, Peter Vauhan, Luke Wheldon, Steven White, Tim White, Imeshi Wijetunga, Michael Wilson, Rebecca Winterborn, Lynda Wyld, Lora Young

**Affiliations:** 1 Centre for Organisational Health and Development, School of Medicine, University of Nottingham, Yang Fujia Building, Jubilee Campus, Wollaton Road, Nottingham, NG8 1BB UK; 2 East Midlands Surgical Academic Network, Queen’s Medical Centre, Nottingham, NG7 2UH UK; 3 Gastrointestinal Surgery, Nottingham Digestive Diseases Centre and National Institute for Health Research (NIHR) Nottingham Biomedical Research Centre, Nottingham University Hospitals and University of Nottingham, Queen’s Medical Centre, Nottingham, NG7 2UH UK; 4 MRC/ARUK Centre for Musculoskeletal Ageing Research, School of Life Sciences, University of Nottingham, Queen’s Medical Centre, Nottingham, NG7 2UH UK

## Abstract

**Background:**

Surgeon burnout has implications for patient safety and workforce sustainability. The aim of this study was to establish the prevalence of burnout among surgeons in the UK during the COVID-19 pandemic.

**Methods:**

This cross-sectional online survey was set in the UK National Health Service and involved 601 surgeons across the UK of all specialities and grades. Participants completed the Maslach Burnout Inventory and a bespoke questionnaire. Outcome measures included emotional exhaustion, depersonalisation and low personal accomplishment, as measured by the Maslach Burnout Inventory-Human Services Survey (MBI-HSS).

**Results:**

A total of 142 surgeons reported having contracted COVID-19. Burnout prevalence was particularly high in the emotional exhaustion (57%) and depersonalisation (50%) domains, while lower on the low personal accomplishment domain (15%). Burnout prevalence was unrelated to COVID-19 status; however, the greater the perceived impact of COVID-19 on work, the higher the prevalence of emotional exhaustion and depersonalisation. Degree of worry about contracting COVID-19 oneself and degree of worry about family and friends contacting COVID-19 was positively associated with prevalence on all three burnout domains. Across all three domains, burnout prevalence was exceptionally high in the Core Trainee 1–2 and Specialty Trainee 1–2 grades.

**Conclusions:**

These findings highlight potential undesirable implications for patient safety arising from surgeon burnout. Moreover, there is a need for ongoing monitoring in addition to an enhanced focus on mental health self-care in surgeon training and the provision of accessible and confidential support for practising surgeons.

**Supplementary Information:**

The online version contains supplementary material available at 10.1007/s00268-021-06351-6.

## Introduction

The eleventh revision of the International Classification of Diseases (ICD-11) conceptualises burnout as an ‘occupational condition’ arising from chronic work-related stress, characterised by being emotionally over-extended and exhausted by one’s work (emotional exhaustion), feelings of cynicism and loss of empathy (depersonalisation), and a sense of low personal accomplishment with respect to one’s work.

Physician burnout has been described as an ‘epidemic’ that affects patient safety, quality of care and patient satisfaction [1]. Burnout can lead to errors in prescribing, reduced quality of medical services, have adverse effects on inter-professional relationships [1–5], and precipitate depression [3] and substance use disorder [6] among medical professionals. It is recognised as a common mental health issue faced by healthcare professionals [1]. Indeed, surgeons have been shown to experience elevated rates of depression and psychiatric distress [7].

Burnout among doctors has been studied to a varying extent, with most of the large studies originating in the USA [4–6, 8]. A survey of 7905 Fellows of the American College of Surgeons found that 40% of respondents were burned out, 30% screened positive for symptoms of depression, and 28% had a mental Quality of Life score > 0.5 standard deviation below the population norm [5]. Only 36% of surgeons felt their work schedule left enough time for personal or family life, and only 51% would recommend that their children pursue a career as a physician or surgeon [5]. Within the same cohort, 8.9% reported concern that they had made a major medical error in the last 3 months, which had an adverse relationship with mental Quality of Life, all three domains of burnout and symptoms of depression.

However, there remains a paucity of contemporary UK-specific evidence. Few UK studies [9, 10] have used the Maslach Burnout Inventory (MBI) [11] that assesses the three domains reflected in the ICD-11 definition and which dominates burnout research, facilitating cross-study prevalence comparisons. Between one quarter and one third of consultant colorectal and vascular surgeons (*n* = 501) surveyed in 2005 reported burnout across the three domains [9], while almost half of consultant surgeons (*n* = 108) surveyed in 2015 reported emotional exhaustion and one quarter depersonalisation [10]. It is likely that the extraordinary pressures of working in the UK National Health Service (NHS) during the COVID-19 pandemic have exacerbated burnout. More than 80% of 141 surgeons from the UK surveyed in 2020 reported having been negatively affected by the pandemic, with reports of fear and anxiety, loss of motivation, low mood, and stress and burnout [12]. Similarly, a survey of more than 4000 NHS employees in 2020 revealed exceptionally high rates of probable common mental disorders (58%) and post-traumatic stress disorder (30%) [13].

This study set out to use the ‘gold standard’ MBI to assess burnout within a large sample of surgeons in the UK during the COVID-19 pandemic. We aimed to establish the overall prevalence of burnout across all three dimensions and identify high-risk groups based on socio- and occupational-demographic characteristics.

## Methods

This was a cross-sectional self-reported online questionnaire study based upon the Human Personnel-specific version of the MBI-Human Services Survey (MBI-HSS) [11] which was run across the UK. The survey was available from 4 January 2021—coinciding with the British Prime Minister’s announcement of a third ‘stay at home’ lockdown order—and closed on the first day of loosening of movement restrictions in England on 29 March 2021. The survey was promoted among consultant surgeons, surgeons in training, associate specialists, and staff and trust grade doctors (non-training) within surgery via social media (Twitter, Facebook and LinkedIn), regional research collaboratives, hospital group e-mails, posters and personal contact. We were not able to calculate the response rate because we do not know how many surgeons saw the invitation to participate. Inclusion and exclusion criteria are listed in Table 1.

**Table 1:** Inclusion and exclusion criteria

Inclusion criteria	Exclusion criteria
Consultant surgeons	Inability to provide informed consent
All grades of surgeons in training (core trainees, specialist trainees and those undergoing specialty fellowships)	Medical students
Staff surgeons	Foundation trainees
Associate specialists	Surgical care practitioners
Trust-grade doctors and Post-Certificate of Completion of Training fellows (all groups aged between 20 and 80 years)	

### Ethics and consent

The study was approved by the University of Nottingham, Faculty of Medicine and Health Sciences Research Ethics Committee (FHMS 485–2002). All participants had to indicate informed consent, having read the participant information sheet on the first page of the questionnaire, by ticking a box at the bottom of the page. Only then could they move on to the main body of the questionnaire.

### Outcomes

The primary outcome was to establish the prevalence of burnout and near-burnout among consultant surgeons and surgeons in training in the UK. Secondary outcome measures were to better understand the risk factors for burnout among consultant surgeons and surgeons in training and to establish the differences in the prevalence of and risk factors for burnout between consultant surgeons and surgeons in training.

### Quantification of burnout

Burnout was measured using the 22-item MBI-HSS (MP) [11] that offers a multi-dimensional assessment of the construct and has been used extensively in surgeon burnout research [14–16]. Three burnout dimensions were assessed: emotional exhaustion, “the problem of lacking sufficient energy to make a useful and enduring contribution at work”, cynicism (depersonalisation), “the difficulty in dealing with other people and activities in the work world”; and personal accomplishment, “the self-evaluation people make regarding the value of their work and the quality of their contribution” [11]. Scores were summed on each burnout dimension, with high emotional exhaustion defined as a score of ≥ 27, high depersonalisation as ≥ 10 and low personal accomplishment as ≤ 33. Consistent with earlier surgeon burnout research [5, 14], burnout was defined as high emotional exhaustion and/or high depersonalisation. Participants were also asked to complete a questionnaire which contained information on participant demographics and established risk factors for burnout (Supplementary Document).

### Statistical analysis

We performed analyses with IBM SPSS version 25 (IBM SPSS, Armonk, NY, USA). The overall prevalence of high burnout in the sample was assessed using descriptive statistics (frequencies and proportions). The prevalence of high burnout was compared across socio- and occupational-demographic groups using Pearson’s Chi-square with 95% confidence intervals.

We generated descriptive statistics for each study variable and applied Pearson’s Chi-square tests to characterise socio- and occupational-demographic related factors and self-reported experiences and perceptions of COVID-19 associated with burnout dimensions: emotional exhaustion, depersonalisation and low personal accomplishment. These variables were dichotomised based on established MBI-HSS cut-off points. We applied Cramer’s *V* to establish effect size, with a coefficient of > 0.10 representing a small effect, > 0.30 a medium effect and > 0.50 a large effect [17]. Statistical significance was defined as *p* < 0.05.

### Data collection and security

The questionnaires were completed online anonymously using the University of Nottingham’s Online Surveys license (https://www.onlinesurveys.ac.uk) which is a secure online survey platform (https://www.onlinesurveys.ac.uk/help-support/online-surveys-security/). The data were collated by the Online Survey platform and populated into a database which was used for analysis. All survey responses were collected over encrypted secure sockets layer (SSL) connections. Access to the collated database was restricted to those personnel approved by the Chief or Local Investigator and recorded as such in the study records.

### Reporting standards

The study was reported in accordance with the guidelines of the STrengthening the Reporting of OBservational studies in Epidemiology (STROBE) statement (https://www.strobe-statement.org) and the American Association for Public Opinion Research (APPOR) reporting guidelines for survey studies [18].

## Results

A total of 621 questionnaires containing responses were submitted. A response rate could not be calculated due to employed sampling strategy. We restricted analyses to respondents who provided complete data across the burnout dimensions (*n* = 601). We assessed remaining cases to ensure data were missing completely at random (MCAR), which was satisfied (Little’s MCAR test, χ^2^ = 293.5, DF = 262, *p* = 0.088). A listwise deletion was utilised to address missing data. This was viewed as an appropriate strategy given that both sample size and MCAR were satisfied [19].

Approximately half of surveyed surgeons reported high levels of emotional exhaustion (*n* = 342, 56.9%) and depersonalisation (*n* = 292, 48.5%), with 14.3% (*n* = 86) indicating low levels of personal accomplishment (Table 2). The prevalence across burnout dimensions (emotional exhaustion, depersonalisation and low personal accomplishment) by socio- and occupational-demographic characteristics is shown in Tables 3 and 4.

**Table 2:** Number of cases among surveyed surgeons across burnout dimensions (n = 601)

	High emotional exhaustion^a^		High depersonalisation^b^		Low personal accomplishment^c^	
	n (%)	95% CI^d^	n (%)	95% CI	n (%)	95% CI
Cases	342 (56.9)	53.2% to 60.6%	292 (48.5)	44.6% to 52.7%	86 (14.3)	11.6% to 17.1%
Total	601		601		601	

^a^High emotional exhaustion = summed score ≥ 27; ^b^High depersonalisation = summed score ≥ 10; ^c^Low personal accomplishment = summed score ≤ 33, ^d^bootstrapped analysis using 1000 sample iteratives to determine 95% CI

**Table 3:** Association between sociodemographic variables and dimensions of burnout

Characteristics (*n*, %)	High emotional exhaustion	High depersonalisation	Low personal accomplishment
*Gender* *(n* = *573)*^*a*^	*n* (%)	*n* (%)	*n* (%)
Female (175, 30.5%)	115 (65.7)	95 (54.3)	34 (19.4)
Male (398, 69.5%)	212 (53.2)	189 (47.5)	50 (12.6)
χ^2^, df, *p* value	7.69, 1, *p* < 0.01	2.25, 1, *p* = 0.109	4.58, 1, *p* < 0.05
*V*	0.116	0.063	0.089
*Age in years* *(n* = *577)*			
≤ 29 (62, 10.7%)	46 (74.2)	45 (72.6)	17 (27.4)
30–44 (277, 48.0%)	156 (56.3)	137 (49.5)	38 (13.7)
45–59 (194, 33.6%)	116 (56.8)	92 (46.7)	25 (12.7)
≥ 60 (41, 7.1%)	11 (26.8)	11 (26.9)	4 (9.8)
χ^2^, df, *p* value	23.05, 3, *p* < 0.001	22.26, 3, *p* < 0.001	9.714, 3, *p* < 0.05
*V*	0.201	0.197	0.129
*Ethnicity* *(n* = *549)*^*b*^			
White (323, 58.8%)	178 (55.1)	157 (48.6)	35 (10.8)
Mixed (17, 3.1%)	13 (76.5)	11 (64.7)	3 (17.7)
Asian/Asian British (109, 19.9%)	63 (57.8)	52 (47.7)	21 (19.3)
Black/African/Caribbean/Black British (16, 2.9%)	7 (43.8)	6 (37.5)	4 (25.0)
Other (84, 15.3%)	48 (57.1)	46 (54.8)	17 (20.2)
χ^2^, df, *p* value	4.15, 4, *p* = 0.533	3.67, 4, *p* = 0.556	9.24, 4, *p* = 0.053
*V*	0.087	0.082	0.130

^a^Five cases removed as the indicated ‘other’ or ‘prefer not to say’; ^b^28 cases indicated they preferred not to say

**Table 4:** Association between occupational demographics and dimensions of burnout

	High emotional exhaustion	High depersonalisation	Low personal accomplishment
Characteristics (*n*, %)	*n* (%)	*n* (%)	*n* (%)
*Grade (n* = *577)*			
CT1-2/ST1-2 (65, 11.3%)	51 (78.5)	48 (73.8)	20 (30.8)
ST3/4 (36, 6.2%)	22 (61.1)	22 (61.1)	7 (19.4)
ST5/6 (53, 9.2%)	37 (69.8)	30 (56.6)	11 (20.8)
ST7/8, Post-CCT Fellow (60, 10.4%)	26 (43.3)	27 (45.0)	2 (3.3)
Non-Consultant Career Grade Doctors (42, 7.3%)	25 (59.5)	22 (52.4)	8 (19.0)
Consultant (321, 55.6%)	168 (52.3)	136 (42.4)	36 (11.2)
χ^2^, df, *p* value	32.6, 7, *p* < 0.001	32.0, 7, *p* < 0.001	29.2, 7,* p* < 0.001
*V*	0.236	0.237	0.225
Specialty (*n* = 571)			
Breast (16, 2.8%)	6 (37.5)	4 (25.0)	1 (6.3)
ENT (16, 2.8%)	8 (50.0)	9 (56.3)	1 (6.3)
General Surgery (263, 45.6%)	158 (60.1)	148 (56.3)	46 (17.5)
Ophthalmology (14, 2.4%)	10 (71.4)	6 (42.9)	3 (21.4)
Plastics (18, 3.1%)	11 (61.1)	8 (44.4)	4 (22.2)
Transplant (29, 0.4%)	13 (44.8)	12 (41.4)	2 (6.9)
Trauma and Orthopaedics (55, 9.5%)	33 (60.0)	29 (52.7)	7 (12.7)
Urology (23, 4.0%)	14(60.9)	9 (39.1)	4 (17.4)
Vascular (87, 15.1%)	42 (48.3)	35 (40.2)	9 (10.3)
Other (55, 10.1%)	33 (60.0)	24 (43.6)	7 (12.7)
χ^2^, df, *p* value	12.1, 13, p = 0.344	20.6, 13, p = 0.064	10.7, 13, p = 0.403
*V*	0.146	0.186	0.136
*Years qualified as doctor (n* = *589)*			
≤ 9 (149, 25.3%)	110 (73.8)	103 (69.1)	37 (24.8)
10–19 (182, 30.9%)	95 (52.2)	81 (44.5)	19 (10.4)
20–29 (132, 22.4%)	75 (56.8)	61 (46.2)	18 (13.6)
≥ 30 (126, 21.4%)	55 (43.7)	42 (33.3)	12 (9.5)
χ^2^, df, *p* value	31.793, 4, *p* < 0.001	39.441, 4, *p* < 0.001	17.782, 4, *p* = 0.001
*V*	0.231	0.256	0.181
*On-call duties (n* = *577)*			
Yes (530, 91.9%)	308 (58.1)	271 (51.1)	81 (15.3)
No (47, 8.1%)	21 (44.7)	14 (30.0)	3 (6.4)
χ^2^, df, *p* value	3.18, 1*, p* = 0.107	7.87,1, *p* < 0.01	2.75, 1, p = 0.096
*V*	0.075	0.118	0.070
*Medical manager (n* = *577)*			
Yes (83, 14.4%)	48 (57.8)	38 (45.8)	10 (12.1)
No (494, 85.6%)	281 (56.9)	247 (50)	74 (15.0)
χ^2^, df, *p* value	0.026, 1, p = 0.990	0.506, 1, p = 0.368	0.491, 1, p = 0.351
*V*	0.012	0.026	0.028

*CCT* certificate of completion of training, *CT* core trainee, *ENT* ear, nose and throat, *ST* specialty trainee

There were significant differences for the prevalence of dimensionality of burnout by some socio-demographic characteristics (Table 3). A higher incidence rate among female surgeons was observed in relation to emotional exhaustion and low personal accomplishment, but not depersonalisation. The effect size ranged from small (emotional exhaustion) to negligible (personal accomplishment). Younger surgeons reported a higher prevalence across all three burnout dimensions than older participants, with what appears to be a decreasing trend within increasing age. The magnitude of these associations was small. Across examined occupational-demographic characteristics, a higher prevalence rate among those in lower grades and with fewer years professional experience since qualifying as a doctor was observed across all three burnout dimensions. The magnitude of this effect was small. In addition, on-call doctors reported a higher level of depersonalisation than those not on-call. This statistical difference was not observed for emotional exhaustion or low personal accomplishment. Differences by speciality or managerial role were not observed. There were significant differences in the rate for burnout by surgeons reported experiences and perceptions of COVID-19 (Table 5). There was no significant difference among those who had or had not contracted COVID-19 across burnout dimensions. However, those surgeons who felt COVID-19 had had a substantial impact on their work were more likely to report prevalence of emotional exhaustion and depersonalisation. The magnitude of this effect was small. Surgeons who reported a high degree of worry about themselves or their family and friends in contracting COVID-19 were more likely to report high emotional exhaustion and depersonalisation and low personal accomplishment. The effect size of was small across all three burnout dimensions.

**Table 5:** Association between COVID-related experiences and perceptions and dimensions of burnout

Characteristics (n, %)	High emotional exhaustion	High depersonalisation	Low personal accomplishment
*Have you had COVID-19?* (*n* = *586*)	*n* (%)	*n* (%)	*n* (%)
Yes (142, 24.2%)	86 (60.5)	67 (47.2)	18 (12.7)
No (444, 75.8%)	247(55.6)	219 (49.3)	68 (15.3)
χ^2^, df, *p* value	1.067, 1, *p* = 0.302	0.197 1, *p* = 0.657	0.599, 1, *p* = 0.439
*V*	0.038	0.020	0.036
*Impact of COVID-19 on work* *(n* = *585)*			
Large (391, 66.8%)	249 (63.7)	211 (54.0)	59 (15.1)
Moderate (150, 25.6%)	65 (43.3)	54 (36.0)	20 (13.3)
Small (44,7.6%)	17 (38.6)	20 (45.5)	7 (15.9)
χ^2^, df, *p* value	25.7, 2, *p* < 0.001	14.2, 2,* p* = 0.001	0.322, 2, *p* = 0.851
*V*	0.213	0.161	0.030
*Worry about getting COVID-19*^*a*^ *(n* = *588)*			
High (51, 8.7%)	39 (76.5)	33 (64.7)	15 (29.4)
Moderate (297, 50.5%)	174 (58.6)	150 (50.5)	43 (14.5)
Low (240, 40.8%)	123 (51.5)	105 (43.8)	28 (11.7)
χ^2^, df, *p* value	11.4, 2, *p* < 0.01	6.3, 3, *p* = 0.019	10.7, *p* < 0.01
*V*	0.135	0.105	0.138
*Worry about friends and family getting COVID-19*^*a*^ *(n* = *588)*			
High (129, 21.9%)	99 (76.7)	87 (67.4)	32 (24.8)
Moderate (399, 67.9%)	208(52.1)	170 (42.6)	46 (11.5)
Low (60, 10.2%)	28 (46.6)	30 (50.0)	8 (13.3)
χ^2^, df, *p* value	27.0, 2, *p* < 0.001	24.1, 2, *p* < 0.01	13.9, 2, *p* = 0.001
*V*	0.209	0.202	0.148

^a^Responses categorised: ‘low’ = “I am not worried…”; moderate = “I occasionally worried…”; high = “I spend much or most of my time worrying …”

## Discussion

To the best of our knowledge, this is the first study to attempt to establish the prevalence of burnout in the UK surgeon population during the COVID-19 pandemic. One quarter of surgeons surveyed in the first three months of 2021 reported having contracted COVID-19. The prevalence of burnout was particularly high on the emotional exhaustion (57%) and depersonalisation (50%) domains, while lower on the low personal accomplishment domain (15%). Burnout prevalence was unrelated to whether respondents had contracted COVID-19; however, the greater the perceived impact of COVID-19 on work, the higher the prevalence of emotional exhaustion and depersonalisation. Degree of worry about contracting COVID-19 oneself and degree of worry about family and friends contacting COVID-19 was positively associated with prevalence on all three burnout domains. Consistent with a survey of National Health Service employees conducted in April-June 2020 which identified higher levels of probable common mental disorders in younger staff, we found an exceptionally high prevalence of burnout in early career surgeons [13].

Contextualisation for the prevalence of burnout during the COVID-19 pandemic observed in our study can be obtained by comparison to pre-pandemic rates among UK surgeons. The only previous study that used the full 22-item MBI in a survey of 501 consultant colorectal and vascular surgeons [9] found prevalence rates of 31–32% for emotional exhaustion and 17–25% for depersonalisation, markedly lower than our study. In contrast, the prevalence of low personal accomplishment (27–31%) was double that observed in our sample. A more recent study involving NHS consultant surgeons (*n* = 108) surveyed in 2015 assessed the emotional exhaustion and depersonalisation dimensions: 44% reported emotional exhaustion and 27% depersonalisation [10]. The equivalent rates for consultants in our study were substantially higher at 52% (emotional exhaustion) and 42% (depersonalisation). While other studies have assessed burnout in UK surgeons, these have used different or abbreviated versions of the MBI, different scoring methods or different measurement instruments altogether [20–24], hindering between-study comparisons. The evolving nature of definitions and approaches to the measurement of burnout presents challenges to its assessment in physician populations [25]. To permit benchmarking and the monitoring of trends within the profession, we recommend that future studies use the MBI-HSS (MP) that reflects the characteristics of the ICD-11 definition of burnout [26] while being tailored to medical professionals.

Comparison with recent pre-pandemic surgeon burnout prevalence data from the UK [10] and that involving multi-country samples [14] indicates the prevalence of emotional exhaustion and depersonalisation was markedly elevated when we assessed it in early 2021 compared with pre-pandemic rates. Such a conclusion is consistent with data indicating that 86% of 141 UK surgeons surveyed felt that they had been negatively affected by the pandemic [12]. An association between the pandemic and low personal accomplishment is more difficult to establish since no recent pre-pandemic UK studies assessed this burnout domain. However, an international study involving 818 surgeons across 86 counties surveyed in 2018 generated a low personal accomplishment prevalence rate of 21%, while the last UK surgeon survey to measure this domain in 2005 generated a prevalence rate of 27–31% across specialities [9]. These rates are higher than the 15% observed in our study, possibly indicating that the COVID-19 crisis and the foregrounding of the contribution of the NHS served to enhance surgeons’ sense of personal accomplishment. Future nationally representative replication studies will help establish whether the prevalence of burnout observed in our study was linked to the pandemic. Ongoing monitoring of trends in burnout will also serve to shine a spotlight on sectors of the profession where intervention may be required.

Our preliminary findings paint a picture of exceptionally high burnout prevalence among trainees who qualified within the last 10 years, and individuals under 30 years of age, with three quarters of respondents in this age bracket reporting high emotional exhaustion and high depersonalisation and one in four reporting low personal accomplishment. High levels of stress-related problems are consistently observed in medical trainee populations internationally [27]. Further research is required to identify the reasons for exceptionally high rates of burnout among these groups in the UK context. We found that the prevalence of burnout across each of the three domains fell markedly in the next age bracket (30–44 years) and years qualified bracket (10–19 years), suggesting that exceptionally high burnout prevalence is an early-career phenomenon. Within the healthcare worker community, surgeons are the least likely group to seek help for mental health problems, possibly owing to concerns about loss of credibility as a doctor and stigma about seeking support within the medical community [28]. These findings are of concern in view of established linkages between burnout and patient safety [29]. The protection and promotion of mental health need to be better addressed in surgeon training to prepare surgeons for a sustainable and fulfilling career. Enhanced theory- and evidence-based interventions are also needed to control burnout across the duration of a career in surgery, with a long-term programme of intervention evaluation and refinement [30].

The strengths of this study lie in use of the widely used MBI that measures burnout as defined by the World Health Organization and ICD-11. Nevertheless, our study has some limitations that must be considered when interpreting the findings. While social media was effective for participant sampling, it hinders response rate calculation. Our findings may not generalise to the surgeon population in the UK owing to the relatively small sample size. Response bias may have been present; surgeons experiencing symptoms of burnout may have been more inclined to participate, leading to their over-representation. Conversely, it has been suggested that surgeons experiencing high levels of burnout may be so overwhelmed that they are less likely to make time for survey completion, resulting in their under-representation [14].

In conclusion, the prevalence of burnout in surgeons assessed during the 2021 ‘stay at home’ lockdown order was elevated relative to pre-pandemic levels, with an exceptionally high prevalence of burnout among early-career surgeons. The findings highlight a need for ongoing monitoring of trends in addition to an enhanced focus on mental health self-care in surgeon training and the provision of accessible and confidential support for practising surgeons. There are important implications for patient care as a burned-out workforce is unlikely to be able to deliver sustained high-quality care.

## Supplementary Information

Below is the link to the electronic supplementary material.

Supplementary file1 (PDF 75 KB)

## Data Availability

Data will be available upon reasonable request from Dr. Jonathan Houdmont (Jonathan.Houdmont@nottingham.ac.uk).
