# Supplementary material for: Burnout Among Surgeons in the UK During the COVID-19 Pandemic: A Cohort Study
Source: World J Surg. 2021 Oct 26;46(1):1–9. doi: 10.1007/s00268-021-06351-6 (PMC8547303; doi:10.1007/s00268-021-06351-6)
Supplement: Supplementary file 1 — Supplementary file1 (PDF 75 KB) [file 268_2021_6351_MOESM1_ESM.pdf]

Page 4: WORK ARRANGEMENTS

5. Grade

6. How many years have elapsed since you qualified as a doctor?

7. Does your job include a regular academic commitment?

8. Do you have a regular private practice commitment?

9. Which speciality do you work in?

More info

- Breast
- Cardiothoracic
- ENT
- General

- Maxillofacial
- Neurosurgery
- Ophthalmology
- Paediatric
- Plastics
- Transplant
- Trauma and Orthopaedics
- Urology
- Vascular
- Other

9.a. If you selected Other, please specify:

10. What type of hospital do you work in?

- Teaching hospital
- District general hospital
- Exclusively in the private sector

11. What proportion of a full working week do you work?

- ≤0.5 full time equivalent
- 0.6-0.7 full time equivalent
- 0.8-0.9 full time equivalent
- 1.0 full time equivalent

12. Do you have on call duties?

- Yes
- No

13. Are you a medical manager?

- Yes
- No

14. Have there been complaints/litigation against you in the last 12 months?

- Yes
- No

Page 5: ABOUT YOU

15. Age

- 20-24
- 25-29
- 30-34
- 35-39
- 40-44
- 45-49
- 50-54
- 55-59
- 60-64
- 65-69
- 70-74
- 75-79

16. Gender

17. Ethnicity

More info

- Bangladeshi (Asian or Asian British)
- Indian (Asian or Asian British)
- Pakistani (Asian or Asian British)
- African (Black or Black British)

- Caribbean (Black or Black British)
- Chinese (Chinese or other Ethnic Group)
- White and Asian (Mixed Heritage)
- White and Black African (Mixed Heritage)
- White and Black Caribbean (Mixed Heritage)
- British (White)
- English (White)
- Irish (White)
- Scottish (White)
- Welsh (White)
- Prefer not to say
- Other

17.a. If you selected Other, please specify:

18. Which region do you work in?

- East of England
- East Midlands
- Kent, Surrey and Sussex
- London
- North East
- North West
- South West
- Thames Valley
- Wessex
- West Midlands
- Yorkshire and the Humber

- Scotland
- Wales
- Northern Ireland

19. How many adults (including you) live within your household?

- 1
- 2
- 3
- 4
- 5
- 6
- ≥7

20. How many children live within your household?

- 0
- 1
- 2
- 3
- 4
- 5
- 6
- 7
- 8
- 9
- 10
- ≥11

21. Do you have carer responsibilities for dependent adult children and/or parents?

- ○ Yes
- ○ No

22. Please describe aspects of your job that you believe may lead to burnout.
Optional

Page 6: THANK YOU

Thank you for taking the time to complete this survey. Your responses have been submitted.

A summary of the study's findings can be obtained from the lead researcher, Professor Dileep Lobo, from January 2021.

If you would be interested in taking part in future research concerning the development of interventions to tackle burnout among surgeons please click the link below. This will take you to a separate form where you may supply an email address.

https://nottingham.onlinesurveys.ac.uk/surgeon-burnout-future-research

---

Key for selection options

4 - In general, how do you find your job?

Not at all stressful
Mildly stressful
Moderately stressful
Very stressful
Extremely stressful

5 - Grade

CT1/ST1 or CT2/ST2
ST3/4
ST5/6
ST7/8
Associate Specialist
Trust-Grade Doctor
Post-CCT Fellow
Consultant

6 - How many years have elapsed since you qualified as a doctor?

≤4
5-9
10-14

15-19
20-24
25-29
30-34
35-39
40-44
45-49
≥50

7 - Does your job include a regular academic commitment?

Yes
No

8 - Do you have a regular private practice commitment?

Yes
No

16 - Gender

Male
Female
Other
Prefer not to say
